# Active environmental surveillance of SARS-CoV-2 in Midwestern United States meatpacking plants

**DOI:** 10.1371/journal.pone.0261704

**Published:** 2021-12-31

**Authors:** Suzanna M. Storms, James F. Lowe

**Affiliations:** 1 Department of Pathobiology, College of Veterinary Medicine, University of Illinois, Urbana, Illinois, United States of America; 2 Department of Veterinary Clinical Medicine, College of Veterinary Medicine, University of Illinois, Urbana, Illinois, United States of America; Beni Suef University Faculty of Veterinary Medicine, EGYPT

## Abstract

This pilot project investigated environmental SARS-CoV-2 presence in seven Midwestern meatpacking plants from May 2020 to January 2021. This study investigated social distancing and infection control practices and incorporated environmental sampling of surfaces and air in employee common areas. All plants increased their social distancing efforts, increased the frequency of cleaning and disinfecting worker areas, and screened for symptomatic people to prevent entry into the workplace. 575 samples from common areas were collected and evaluated with RT-qPCR for the presence of SARS-CoV-2. 42/367 surface samples were positive, while no virus was detected in air samples. Case positive data from the counties surrounding each plant showed peak positive SARS-CoV-2 cases from 12–55 days before the virus was detected in the plant, indicating that environmental sampling is likely a lagging indicator of community and plant infection.

## Introduction

Data evaluating the airborne and surface transmission routes of severe acute respiratory syndrome coronavirus-2 (SARS-CoV-2) are limited and are based on previous work with other respiratory viruses [1]. Airborne droplets are thought to be the most likely source of transmission of respiratory viruses, but evidence of surfaces as an indirect route of transmission for SARS-CoV-2 has been shown [2]. Nasopharyngeal aspirates as well as stool samples of SARS infected patients showed high viral loads, which may facilitate surface transmission of virus in common areas or on fomites in the environment [3]. SARS-CoV-2 aerosols have a half-life of 1 hour for infectious virus [4]. In contrast, studies of the viability on surfaces for SARS-CoV-2 and the related SARS and MERS corona viruses demonstrate a variable period of viability on surfaces depending on the surface type where plastic has the longest half-life of 7-hours [1, 4]. The prolonged and variable half-lives of these viruses suggest that surfaces may pose a considerable risk of indirect transmission to susceptible populations. Mask wearing and social distancing are the frontline defenses against airborne transmission, and effective cleaning and disinfection is essential to prevent surface transmission of coronaviruses [5].

Little is known about SARS-CoV-2 transmission in meatpacking facilities. Meat and poultry processing facilities are considered essential critical infrastructure within the Food and Agriculture Sector by the US Department of Homeland Security [6]. During the spring and early summer of 2020, more than 60 commercial meatpacking facilities across the United States reduced operations related to COVID-19 [7]. According to the Health and Safety teams at the plants, the workplaces faced absenteeism from sick employees, and workers feared going to work due to the risk of disease in the workplace. Because of the slaughter chain’s reduced capacity, an estimated 3.4 million hogs and 1.3 million cattle were unable to be processed on time, leading to a backlog of animals, and creating problems for producers in all stages of swine and cattle production [8].

To learn more about SARS-CoV-2 presence in packing plants, possible routes of transmission to essential workers, and ways to surveille and prevent further infections, we partnered with seven finished pig meatpacking plants in the Midwestern United States. Our parameters for the Midwestern United States are defined by the USDA Farm Resource Regions provided by the Economic Research Service and encompasses the Heartland Region [9]. This study aimed to detect the plants’ new infection control practices and determine the environmental SARS-CoV-2 presence in the plants on surfaces and the air. Plants were sampled on a weekly basis in high traffic areas, and sites were identified in the plants that were routinely contaminated with SARS-CoV-2. No virus was identified in air samples. Despite health screening of employees, all plants participating in the winter of 2020 tested PCR positive for surface contamination with SARS-CoV-2.

## Materials and methods

The study was divided into two phases. Phase 1 took place from May to July 2020 and followed plants A and B weekly with surface, air, and observational study. Lab members aided in sample collection and observation. Phase 2 took place from September 2020 until January 2021. In this phase, five additional plants of varying sizes were followed. Phase 2 only included surface and air sampling. Health and safety teams from each plant collected and shipped samples to our lab according to written protocols.

### Plant observation

In phase 1 of the trial, plants A and B were each observed weekly for 4 and 5 weeks respectively. Observations were made on increased hygienic practices, face mask and face shield compliance, and other social distancing practices. Cafeterias, restrooms, office spaces, and the production floor were observed. We worked closely with the plants’ health and safety teams to understand what new safety and preventative measures were being implemented in the plants. In phase 2 of the trial, plants were not observed, and no information about preventative measures was taken.

### Surface sampling

The study’s second objective was to measure the presence of SARS-CoV-2 on surfaces in the plants’ common areas. We targeted high touch areas, such as the lunchrooms, vending machines, microwaves, restrooms, locker rooms, office spaces, doorknobs, time clocks, among others. We sampled 179 surfaces over seven weeks in plants A and B in phase 1 of the study.

During phase 2 of the study, samples were collected from plants B to G and placed in 2mL PrimeStore MTM for shipment to the lab [10]. In phase 2 of the trial, 367 surfaces were sampled in the same high-touch areas.

Environmental samples were obtained using a dry electrostatic cloth (Swiffer, Proctor & Gamble, Cincinnati, OH), cut into eighths (67.2cm2) and the cloth wiped on a surface or a combination of high touch surfaces (such as all vending machine buttons in one room) and placed into a 15mL tube containing 5mL of BHI viral transport media [11, 12]. Gloves were changed between each sample. High touch surfaces were sampled in the cafeterias, restrooms, hallways, and office spaces. Samples were placed with ice packs and chilled until further processing at the lab. During the second phase of the study, samples were placed into a 15mL tube containing 2mLof PrimeStore MTM (Longhorn Vaccines & Diagnostics, LLC, Bethesda, MD, USA), an FDA approved media and tube system used for nucleic acid storage, stabilization, and transportation device. PrimeStore MTM can be shipped through standard mail and does not require refrigeration of the samples [13]. Samples were processed at the lab by briefly vortexing each tube and eluting the transport media from the cloth. The eluate was submitted to the University of Illinois Veterinary Diagnostic Lab for RT-qPCR. RNA purification was performed with 100 μL of each environmental sample by using the BioSprint 96 One-For-All Vet kit (QIAGEN, Germantown, MD USA) and a Kingfisher 96 instrument (Thermo Scientific, Waltham, MA, USA). Viral RNA was eluted into 75 μL of buffer. Real-time reverse transcription PCR (rRT-PCR) was performed on the nucleic acid extracts using the CDC 2019-Novel Coronavirus Real-Time RT-PCR Diagnostic Panel kit amplifying the N1 or N2 protein according to the manufacturer’s recommendations [14].

### Air sampling

Similarly, we tested the air in these same areas of worker concentration, with most samples taken in lunchrooms and locker rooms. Due to the large air turnover on the packing floor, which is greater than eight air turns per hour, we sampled areas of high worker concentration in locations in the plant with normal commercial or residential air handling systems. We first sampled air during busy lunch break periods, when many workers were present in the rooms, with their masks lowered for eating and drinking, collecting 6000L of air per sample. We later began sampling for 24 hours collecting 144,000L samples, allowing time for all plant workers to move through the rooms during a 24-hour period. Twenty-nine 30-minute air samples were taken in phase 1 of the trial, and twenty-two 24-hour samples were taken in phase 2 of the trial.

Air samples were taken using air collectors designed and built for this project. Each air collector housed a negative pressure fan powered by a 24v rechargeable battery. The fan sampled room air across a Bobcat Rapid Filter Elution PBS Kit filter (Innovaprep, Drexel, MO, USA) at a speed of 200L/min according to the manufacturer’s instructions for SARS-CoV-2 sampling. In phase 1 of the study, filters were eluted with 5mL PBS provided in the kit according to manufacturer’s instructions. In phase 2 of the study, filters were not eluted, but placed directly into the PrimeStore MTM tube for virus stabilization and transport back to the lab.

### Data analysis

Data was collected from PCR results run at the University of Illinois Veterinary Diagnostic Lab. PCR results from surface and air samples were compiled by date, plant, sample type, and location samples were taken. Percentages were calculated for PCR samples, with total samples collected as the denominator, and PCR positive samples as the numerator. Percentages were displayed as percent positive per plant, per week.

## Results

### Increased hygiene

In phase 1, all plants implemented temperature checks for all employees before entering the plant, enforced mandatory face masks, and Plant B required face shields. Both plants hired more cleaning crews, and Plant B did small droplet chlorine dioxide disinfection of different employee spaces during the night. Plant B also installed UV light systems in the ductwork supplying the main cafeteria, with plans to install more for the locker room air handling systems. Plant B installed foot pulls or propped open all doors to decrease hand contact on doorknobs and installed hand sanitizing stations at all entrances to the plant and cafeteria.

### Social distancing

In phase 1, both plants made changes to increase social distancing. In both plants, tables were cleaned between each employee and temporary partitions, with plans for permanent partitions, were placed on the production floor between workers. In addition, Plant B renovated spaces to double the size of their cafeteria space, installed plexiglass partitions at lunch tables to prevent droplets from spreading across or next to the person, and installed more time clocks throughout entry to the plant to decrease time spent congregating. Managers were present to enforce both the six-foot distance between employees and mask and face shield compliance in common spaces. Because of limited space, some of the plants included in phases 1 and 2 of the trials expanded outdoor eating spaces to lower the density of workers indoors during breaks. However, Plant A did not enforce the mask policy as strictly as Plant B, or the social distancing during break times or while on the production floor as rigorously as Plant B.

### Surface and air sampling

**Fig 1:**
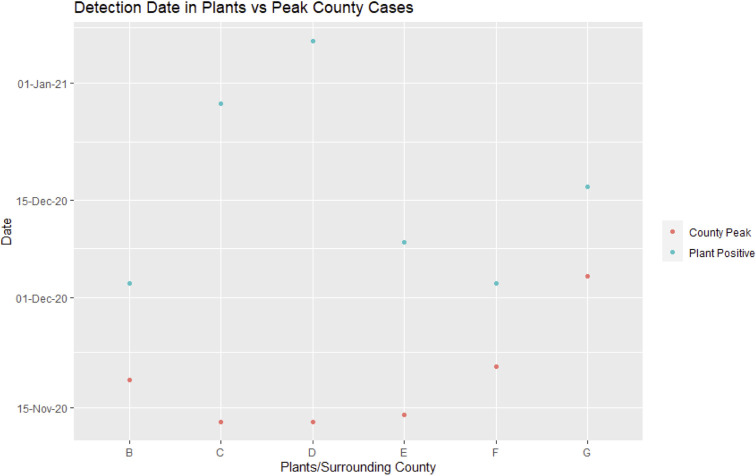
SARS-CoV-2 detection in plants vs peak county cases. Each letter on the x-axis represents a meatpacking plant and its surrounding county. Plotted on the y-axis are the dates that each county peaked with SARS-CoV-2 positive tests and the date that SARS-CoV-2 was detected in each plant by our surveillance [15–18].

SARS-CoV-2 was not detected in surface or air samples by rRT-qPCR in phase 1 of the study (Table 1). In phase 2, SARS-CoV-2 was detected in surface samples from all plants. SARS-CoV-2 presence began to be detected the week beginning on 11/29/2020. Samples continued to test positive for the weeks following, with all six plants in phase 2 testing positive at least one time (Fig 1, Table 2). Plants, dates, surfaces, and air sampled, as well as positive CT values, are shown in the S1 Appendix.

**Table 1:** Phase 1 surface and air sample collection.

Week of 2020	Plant A	Plant B
5/25/20	0/33	n/a
6/8/20	0/13	n/a
6/15/20	0/23	0/15
6/22/20	0/22	0/16
6/29/20	0/24	0/21
7/6/20	0/21	0/20
Total	0/136	0/72

Air and surface samples collected by week from Plants A and B during phase 1 of the study. Numerator indicates positive samples and denominator indicates total number of samples collected per week. No air or surface samples were detected positive in phase 1 of the study. 208 samples were collected in phase 1 of the study.

**Table 2:** Phase 2 surface and air sample collection.

Week of 2020	Plant B	Plant C	Plant D	Plant E	Plant F	Plant G
9/14/20			0/15			
9/21/20			0/7			
9/28/20			0/8			
10/5/20						
10/12/20						
10/19/20			0/9			
10/26/20		0/6	0/8			
11/2/20		0/6				
11/9/20		0/7	0/14			
11/30/20	3/7				3/5	
12/7/20	9/14			1/7	0/9	
12/14/20	10/69				0/10	3/8
12/21/20	4/17				0/1	
12/28/20	2/6	1/7	0/6		0/10	1/8
1/4/21	0/6	0/7	2/7	1/13	0/10	0/8
1/11/21		1/8	1/4	0/6		0/8
1/18/21		0/8				0/8
Total	28/119	2/49	3/88	2/26	3/45	4/40
	(23.5%)	(4.1%)	(3.4%)	(7.7%)	(6.7%)	(10%)

Air and surface samples collected by week from Plants B-G during phase 2 of the study. Numerator indicates positive RT-qPCR detection of SARS-CoV-2. Denominator indicates total number of samples collected per week. All positive samples were collected from surfaces. Total numbers show percentage positive samples of all samples collected from each plant. SARS-CoV-2 was detected on surfaces in all plants by the end of the study. 367 samples were collected during phase 2 of the study.

At the end of the study period, 42/367 surface samples were detected positive for SARS-CoV-2 RNA. In general, positive samples were from cafeteria surfaces, such as tables, microwave buttons, or vending machines. CT values in the 20s were also detected in restroom surface samples and on office surfaces. Several areas such as handrails, maintenance equipment, and health services had CT values in the upper 30s near the positive cut off value. Overall, most positive samples had CT values in the high 30s indicating only low amounts of SARS-CoV-2 RNA. No SARS-CoV-2 was detected in air samples. After identifying problem areas, health and safety teams focused attention on those areas to decrease the viral load on surfaces with a combination of disinfection and detergent cleaners.

## Discussion

SARS-CoV-2 surveillance in packing plants was one part of a larger integrated strategy to protect essential workers from COVID-19 and to ensure a steady supply of meat to the American consumer. Samples collected from the environment where workers congregate during breaks was an efficient and low-tech method to sample for presence of virus in the plant, however, it was a reactive testing strategy.

We did not detect coronavirus in air samples, leading us to believe that the potential for airborne transmission in the plant with current COVID-19 mitigation strategies is low. This could be attributed to daily health screening and excluding symptomatic employees from entering each plant, as well as implementing mandatory masks, face shields, and the expanded or new outdoor spaces used when facemasks were lowered for eating. Also, air changes on the packing floor (work areas) are high due to the sanitary requirements to prevent bacterial contamination of meat, although many of the breakroom areas in the studied plants have conventional HVAC systems. Testing for viral particles in air samples is challenging, especially when there are no acute or symptomatic employees present in the plants. Because of the mitigation practices preventing symptomatic employees from entering the plants, mandatory face masks, partitions at lunch tables, partitions on the packing floor, UV lights in the HVAC systems, and face shields in some plants, we believe that the risk of transmission of virus via inhalation is low.

We found that environmental testing in packing plants was a lagging indicator of SARS-CoV-2 detection in the plants. Positive cases reported at the county level rose prior to positive environmental sample detection in the two plants (C and D) that were surveilled prior to any positive tests in the plants (Fig 1) [15–17]. Environmental sampling did not enable us to predict an increase in cases and therefore precluded our ability inform the plant to heighten cleaning procedures before the number of cases in the community increased.

SARS-CoV-2 was not detected in phase 1 of the study, suggesting that the plants’ workforce was healthy during the May-July 2020 sampling period, with symptomatic workers staying home. Cases in the surrounding communities were also quite low during this period [17]. As cases rose in the communities surrounding the plants, around the Thanksgiving holiday at the end of November 2020, environmental surface sample detection in the plant also increased [18]. No in-person observations of plant mitigation procedures were made by our lab during the winter of 2020, but plants reported daily health screening and questionnaires prior to employee entry in order for only non-febrile, asymptomatic workers to enter the facilities. The detection of SARS-CoV-2 RNA in the plant where only asymptomatic workers were present concurs with the plethora of information published about the occurrence of asymptomatic and presymptomatic infections with ranges from 4–41% of infected individuals showing no symptoms [19]. This study corroborates that asymptomatic employees were shedding virus on surfaces in every plant surveilled in winter 2020.

A limitation for this study is that only RNA was detected, and the amount of viable, infectious virus in the samples is unknown. A CT value of 38 may or may not be infectious virus; it may be fragments of RNA left behind after proper disinfection [20–22]. Another limitation of our study was the inability to observe the plants in phase 2 of the study. We are unable to comment on how their hygienic practices or social distancing impacted the CT values reported for their surface samples. The plants established strict no-visitor policies during phase 2 of the study preventing our observation of plant practices. We are very appreciative of the health and safety teams who collected and sent us samples and were outstanding partners in helping us understand what was happening in the plant, both with biosafety practices and worker health. The health and safety teams in the packing plants are well trained in foodborne pathogen prevention and in preventing physical injuries to the workforce but had a steep learning curve to implement human infectious disease preventative measures given the ambiguity of the best control practices for SARS-CoV-2 early in the pandemic.

## Conclusion

This study highlights the value of ongoing, prospective monitoring of workplaces for infectious disease and the value of academic-industry partnerships to address complicated, real-world problems. It also demonstrates that routine monitoring in the meatpacking plants with asymptomatic workers was likely a lagging indicator of community SARS-CoV-2 infections. This suggests that systematic prospective monitoring in the community is more likely to be successful in protecting worker health in the workplace than a workplace-based sampling strategy. Developing this infrastructure, especially in underserved communities, is an important goal to address community and worker health.

## Supporting information

S1 Appendix (XLSX)
